# Development and validation of a horse reference panel for genotype imputation

**DOI:** 10.1186/s12711-022-00740-8

**Published:** 2022-07-04

**Authors:** Paula Reich, Clemens Falker-Gieske, Torsten Pook, Jens Tetens

**Affiliations:** 1 Department of Animal Sciences, Georg-August-University Göttingen, 37077 Göttingen, Germany; 2 Center for Integrated Breeding Research (CiBreed), Georg-August-University Göttingen, 37075 Göttingen, Germany

## Abstract

**Background:**

Genotype imputation is a cost-effective method to generate sequence-level genotypes for a large number of animals. Its application can improve the power of genomic studies, provided that the accuracy of imputation is sufficiently high. The purpose of this study was to develop an optimal strategy for genotype imputation from genotyping array data to sequence level in German warmblood horses, and to investigate the effect of different factors on the accuracy of imputation. Publicly available whole-genome sequence data from 317 horses of 46 breeds was used to conduct the analyses.

**Results:**

Depending on the size and composition of the reference panel, the accuracy of imputation from medium marker density (60K) to sequence level using the software Beagle 5.1 ranged from 0.64 to 0.70 for horse chromosome 3. Generally, imputation accuracy increased as the size of the reference panel increased, but if genetically distant individuals were included in the panel, the accuracy dropped. Imputation was most precise when using a reference panel of multiple but related breeds and the software Beagle 5.1, which outperformed the other two tested computer programs, Impute 5 and Minimac 4. Genome-wide imputation for this scenario resulted in a mean accuracy of 0.66. Stepwise imputation from 60K to 670K markers and subsequently to sequence level did not improve the accuracy of imputation. However, imputation from higher density (670K) was considerably more accurate (about 0.90) than from medium density. Likewise, imputation in genomic regions with a low marker coverage resulted in a reduced accuracy of imputation.

**Conclusions:**

The accuracy of imputation in horses was influenced by the size and composition of the reference panel, the marker density of the genotyping array, and the imputation software. Genotype imputation can be used to extend the limited amount of available sequence-level data from horses in order to boost the power of downstream analyses, such as genome-wide association studies, or the detection of embryonic lethal variants.

**Supplementary Information:**

The online version contains supplementary material available at 10.1186/s12711-022-00740-8.

## Background

Genomic studies that use data from single nucleotide polymorphism (SNP) arrays are based on the linkage disequilibrium (LD) between markers and causal variants [1, 2]. In contrast, whole-genome sequence (WGS) data potentially include all causative mutations [3], which means that applications using WGS data are independent from LD structures [4]. Accordingly, using sequence data is expected to improve the power of genome-wide association studies (GWAS) and genomic selection (GS) [5]. In GWAS, the benefit of using WGS data is obvious: while the use of SNP array data allows mapping causal variants to a genomic region, the exact identification of the causative mutations requires sequence data [6]. The benefit of using WGS data in GS is somewhat controversial and has not necessarily been proven to improve its accuracy [7, 8]. Nevertheless, WGS data can be used to indirectly increase the accuracy of GS by completing commercial SNP arrays with variants selected from WGS based on GWAS [9, 10], or by developing customised SNP arrays with markers that are specifically selected for informativity [6].

However, to be beneficial, WGS data have to be available for a large number of individuals but sequencing large cohorts of animals is still expensive [11]. A less cost-intensive alternative to obtain sequence data for a large number of animals is to impute from lower marker density to sequence level [5]. Genotype imputation is the prediction of genotypes that have not been directly assayed in a study sample using a reference panel of haplotypes [12, 13]. By increasing the number of markers that are available for association testing, genotype imputation can boost the power of GWAS, facilitate meta-analyses, and improve fine-mapping of causative variants [12], e.g., because genetic variation that was not previously explained by the data can be included [14]. However, inaccurate imputation can affect the results of subsequent analyses [11]. Therefore, evaluating the accuracy of imputation is useful, to make the exclusion of poorly imputed markers from further investigations possible [1].

Several factors affect the accuracy of imputation, among which the software applied for imputation [15, 16], minor allele frequency (MAF) of the imputed markers [17, 18], marker density of the SNP array used for genotyping the study samples [19, 20], as well as the size and composition of the reference panel [21, 22]. Increasing the number of animals in the reference panel generally improves the accuracy of imputation, but including genetically distant animals can also result in higher error rates [1, 23]. A higher marker density of the genotyping array increases the accuracy of imputation, which can be further improved by stepwise imputation, first to an intermediate, and then to the final density of interest [5, 20].

Only a few studies have investigated the accuracy of imputation in horses [24–28]. German warmblood horses represent a large horse population of worldwide importance, but the amount of information available on imputation accuracy is very limited for this breed while the implementation of GS is a declared aim [29]. Therefore, the purpose of this study was to develop an optimal strategy for genotype imputation from SNP array data to sequence level in German warmblood horses, and to investigate the impact of different factors on the accuracy of imputation.

In addition, our aim was to characterise the variants present in the publicly available WGS data from various horse breeds that were used for the imputation study, and use these data to detect putative genetic defects based on missing homozygosity. Recessive mutations that are lethal in utero can be identified from variants that are present in a population at rather high frequencies, but never occur in the homozygous state in live individuals [30]. This method was first applied in cattle a decade ago [30]. Since then, based on the principle of missing homozygosity, several potentially deleterious haplotypes have been discovered in cattle (e.g., [31–34]) and other livestock, such as pigs [35, 36].

In horses, this approach to identify potentially lethal mutations has only been employed to a limited extent. Schrimpf et al*.* [37] scanned the equine sequence data of genes that are predicted to be involved in male reproduction for high-impact variants showing missing homozygosity. They detected a couple of variants that potentially influenced stallion fertility, including a splice-site disruption variant in the *NOTCH1* gene [37]. Furthermore, a haplotype in the *LY49B* gene was detected from genotype data on Thoroughbreds that showed a deficiency of homozygotes, and it represents a strong candidate for containing a recessive lethal mutation [38].

The detection of lethal variants can also aid in diminishing the prevalence of genetic disorders and in the optimisation of fertility rates in horses [38, 39]. Therefore, we applied an approach based on missing homozygosity to identify putative lethal mutations. Within the limited and diverse panel of sequenced horses used here, this yielded no statistically significant results. However, these additional analyses illustrate some of the possible applications of WGS data from horses, which could be obtained for a much larger number of animals by applying genotype imputation to boost the power of future studies.

## Methods

### Data collection, mapping and variant calling

Publicly available WGS data on 360 horses from 50 breeds were obtained from the European Nucleotide Archive (ENA) at EMBL-EBI. According to the information available in the NCBI Sequence Read Archive (SRA), all animals were sequenced using Illumina paired-end sequencing technologies and the average sequencing coverage was 26.3-fold (range 5.0 to 212.8). A detailed list of all horse genomes used in the study is in Additional file 1: Table S1.

Mapping and variant calling were performed using the genome assembly EquCab3.0 (GCA_002863925.1, Ensembl release 100) and the Genome Analysis Toolkit (GATK) version 4.1.7.0 [40] following the GATK best practices recommendations [41]. Briefly, conversion of fastq to bam files and addition of read group information was achieved with the tool FastqToSam before marking Illumina adapters with MarkIlluminaAdapters. Reads were mapped to the reference genome using the SamToFastq, BWA-MEM version 0.7.12 software [42] and MergeBamAlignment. MarkDuplicates was applied to mark duplicates and technical artefacts, and base quality scores were recalibrated using BaseRecalibrator and ApplyBQSR. The known-sites dataset for Base Quality Score Recalibration (BQSR) was dbSNP build 151. Variant calling was performed per sample with HaplotypeCaller in ERC GVCF mode and the resulting GVCF files were merged with CombineGVCFs and jointly genotyped with GenotypeGVCFs. Variant filtering of the raw variants was performed separately for SNPs and insertions and deletions (INDELs). SNPs were hard-filtered on the ExcessHet annotation (filter setting: ExcessHet > 54.69) with VariantFiltration before applying Variant Quality Score Recalibration (VQSR) with the VariantRecalibrator and ApplyVQSR tools. A transition/transversion (Ti/Tv) free recalibration was performed because of the use of two truth datasets for VQSR: the variants of the EquineSNP50 BeadChip (Illumina) and the GeneSeek Genomic Profiler (GGP) Equine BeadChip (Neogen/Illumina), which were successfully lifted over to EquCab3.0. For the liftover, Illumina SNP array ID were converted to dbSNP rsID using SNPchiMp v.3 [43] and positions were acquired from the EquCab3.0 dbSNP build 151 with SelectVariants. The dbSNP build 151 was the known sites’ resource applied for VQSR and the truth sensitivity was set to 99.0. Lacking an independent and reliable truth dataset for their filtering, INDELs and mixed-type variants were hard-filtered with VariantFiltration using the following filter settings: QD < 2.0, QUAL < 30.0, FS > 200.0, ReadPosRankSum < -20.0 and ExcessHet > 54.69.

The first rounds of subsequent analyses revealed the inadequacy of several horse genomes from three projects, which were excluded from further studies, as specified in Additional file 1: Table S1. The final dataset used for all following analyses consisted of 317 horse genomes from 46 breeds with an average sequencing coverage of 21.4 (range 5.0 to 67.4). Variant statistics were produced with BCFtools stats version 1.9 [44].

### Preparation of reference panels for imputation

To determine the genetic relationship of the 317 horses in the variant call set, multidimensional scaling (MDS) was performed using the PLINK 1.9 software [45] and R version 4.0.5 [46] for plotting the data. The analysis was carried out based on the marker positions of the GGP Equine Plus BeadChip (Neogen/Illumina) that contains 71,947 markers. After lifting them over to the new genome assembly EquCab3.0, 61,559 of the variants were present in the variant call set and available for the analysis.

Based on the results of the MDS plot, the horses were assigned to three groups depending on their genetic relationship to German warmblood horses, in order to define three versions of the reference panel for imputation, which differed in size and breed composition. Reference panel 1 (RP1) only included the most closely related animals forming the first group and therefore had the smallest number of horses. Reference panels 2 (RP2) and 3 (RP3) contained progressively more animals that were increasingly less related. This was achieved by first adding the second (RP2) and then the third group of horses (RP3) to RP1.

### Imputation procedure and estimation of imputation accuracy

Haplotype phasing of the variant call set of 317 horses was performed using Beagle 5.1 [47] with default parameter settings, and the effective population size (N_e_) set to 1000. Likewise, genotype imputation for the pre-phased data was carried out using Beagle 5.1 [48] with standard settings and N_e_ set to 1000 unless stated elsewhere.

All imputation procedures were performed for *Equus caballus* chromosome 3 (ECA3). The imputation scenario resulting in the highest accuracy of imputation was further applied to the whole genome. Imputation accuracy was evaluated using five-fold cross-validation in 65 warmblood horses and closely-related breeds (horses from RP1). Individuals were divided into five equally-sized groups (a) to (e) of 13 animals and each group was used once for validation. For the animals to be imputed, all genotypes were masked, except for the aforementioned markers on the GGP Equine Plus BeadChip (Neogen/Illumina) or the markers on the Axiom Equine Genotyping Array (Axiom MNEc670; Applied Biosystems) for imputation from medium (60K) or high marker density (670K), respectively. The number of variants on ECA3 that were available for the imputation was 2959 for the medium-density array and 25,925 for the high-density array, while the sequence data contained 1,382,365 variants. Imputation for all autosomes from medium-density to sequence level was performed from 58,713 markers to 26,631,480 variants. All splitting and masking procedures of the phased variant call set were carried out using the tool SelectVariants from GATK version 4.1.8.1 [40].

Imputation accuracy (*r*) per SNP was calculated as the correlation between observed genotypes and estimated alternate allele dosages across all samples. For this purpose, the datasets were converted to additive coding with PLINK 2.0 [45] and the accuracy of imputation was calculated with an in-house R script (see Additional file 2) using R version 4.0.5 [46]. Multi-allelic sites as well as variants that showed no variation for the observed genotypes or the estimated alternate allele dosages in at least one of the validation groups were excluded from the analysis. Minor allelic frequency for the variants was calculated from the sequence data of all animals included in RP1.

### Imputation scenarios

To study the effect of different factors on the accuracy of imputation, and to determine an optimal strategy resulting in the highest imputation accuracy for warmblood horses, several scenarios were considered for imputation. Three reference panels differing in size and breed composition (RP1, RP2 and RP3) were used to assess the impact of the number of sequenced individuals and their genetic relationship with the study samples on the accuracy of imputation. In all three cases, the target panel for imputation was one-fifth of RP1, hence including 13 animals. The reference panel consisted either of the remaining four-fifths of RP1 (imputation with RP1; 52 animals), the remaining four-fifths of RP1 plus all the animals in RP2 (imputation with RP2; 162 animals), or the remaining four-fifths of RP1 plus all the animals in RP2 and RP3 (imputation with RP3; 304 animals). Imputation was carried out from medium marker density to sequence level. For the scenario resulting in the best accuracy of imputation, the analysis was repeated using two additional software tools, Impute 5 [13] and Minimac 4 [49]. Impute 5 was run with default parameter settings and N_e_ set to 1000. Since the adjustment of N_e_ is not supported in Minimac 4, the program was run without any modifications of the default settings. The reference panel was converted to M3VCF format including parameter estimation using Minimac 3 [49] before performing imputation with Minimac 4. Since the use of these two software tools could not improve the accuracy of imputation, all subsequent analyses were conducted using Beagle 5.1.

To clearly separate the effect of the size of the reference panel from the effect of breed composition, two additional scenarios were studied for RP1. Instead of using all remaining four-fifths as the reference group, only the following two- or three-fifths were considered (e.g., for validation group (c), the groups (d) and (e) or (d), (e) and (a) were included in the reference group).

To investigate the impact of the marker density of the SNP array used for genotyping the study samples on imputation accuracy, the procedure for all three reference panels was repeated for imputation from high marker density to sequence level. Furthermore, a two-step approach for RP1 was assessed to analyse if a stepwise imputation could improve the accuracy of overall imputation. In step one, imputation was carried out from medium to high marker density using the previous two-fifths of the respective validation group as the high-density reference panel (e.g., group (e) and (a) for validation group (b)). For the high-density reference panels, all markers except those on Axiom MNEc670 were masked. In step two, imputation was performed from the high-density marker data obtained in step one to sequence level using the following two-fifths of the validation group as the sequenced reference panel (e.g*.*, group (c) and (d) for validation group (b)). In an additional scenario, step two of the imputation was performed from only the markers obtained in the first step that had a Dosage R-Squared (DR2) value greater or equal to 0.4. Step one, i.e., imputation from medium- to high-density, was subsequently repeated including all remaining four-fifths of RP1 in the high-density reference panel.

### Analysis of information content

In addition, we investigated the proportion of variation in the 60K data, the imputed panel and the sequence data that could be explained by the variation in the other two respective datasets. For this, we used a Monte Carlo analysis of variance as suggested by de los Campos et al*.* [14]. For the imputed panel, two scenarios were considered. First, the panel was created by combining the five panels with 13 animals each that were separately imputed. Second, the panel was created by performing imputation on the full set of 65 horses of RP1 jointly and using only the remaining 252 animals of RP3 as the reference panel.

### Variant effect prediction

To analyse the likely impact of the identified variants, and to detect putative lethal mutations, variant effect prediction was performed for the reduced variant call set of 317 horses using SnpEff version 5.1 [50] with default settings and the genomic database EquCab3.0.99. Multi-allelic sites were excluded from the dataset prior to conducting the analysis. Variants that were predicted to have a high impact on protein-coding sequences were filtered out using SnpSift version 5.1 [51]. These variants were tested for a significant absence or reduction of homozygotes under the assumptions of Hardy–Weinberg equilibrium using a binomial test in R version 4.0.5 [50]. The obtained p-values were adjusted for multiple testing using Bonferroni correction.

## Results

### Variant discovery

The analysis of WGS data from 317 horses identified 27,685,397 polymorphic sites, of which 1,496,735 were multiallelic. The total numbers of SNPs and INDELs were 24,540,424 and 5,025,312, respectively. Among these variants, 17,323,823 (58.6%) were previously known, i.e., 70.1% of the SNPs (17,212,051) and 2.2% of the INDELs (111,772). The Ti/Tv ratio for SNPs was 1.98, and 2.04 for known and 1.85 for novel sites.

### Composition of reference panel groups

**Fig. 1:**
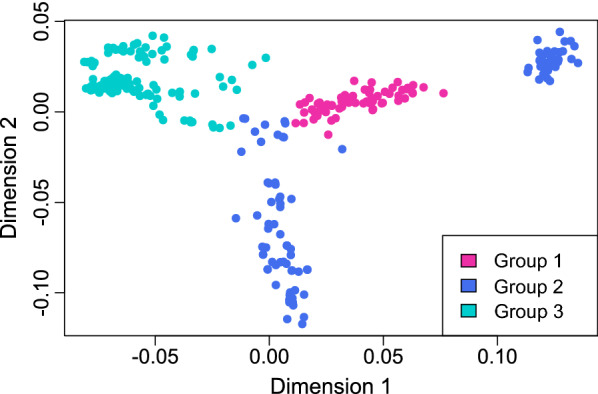
Multidimensional scaling plot showing the genetic relationships among the 317 horses included in this study. Based on the results of the plot, the horses of our variant call set were assigned to three groups, depending on their genetic relationship to German warmblood horses, in order to determine different versions of the reference panel used for imputation. The localisation of all German warmblood horses in the purple cloud of points served as the basis for assigning all other individuals to their respective groups

In accordance with the results of the MDS plot (Fig. 1), the horses were assigned to three groups depending on their genetic relationship to German warmblood horses. All animals that clustered directly with the warmblood horses were assigned to group 1. Overall, this group included 65 individuals that belonged predominantly to different breeds of German warmblood horses and Quarter horses. Individuals that clustered closely with the warmblood horses were assigned to group 2, which was composed of 110 horses that were mostly Arabians, Thoroughbreds and Standardbreds. Group 3 included the remaining 142 horses that belonged to various breeds.

These three groups were used to define three versions of the reference panel (RP) for imputation that differed in size and breed composition. RP1 consisted only of the individuals of group 1, RP2 included the individuals of group 1 plus those of group 2, and RP3 contained the horses of all three groups. Thus, the three versions of the reference panel used for genotype imputation contained 65 (RP1), 175 (RP2) and all 317 horses (RP3), each without 13 animals from RP1 that formed the target panel for imputation in the cross-validation scheme. The individual classification of all horses is in Additional file 1: Table S1.

### Accuracy of imputation

**Fig. 2:**
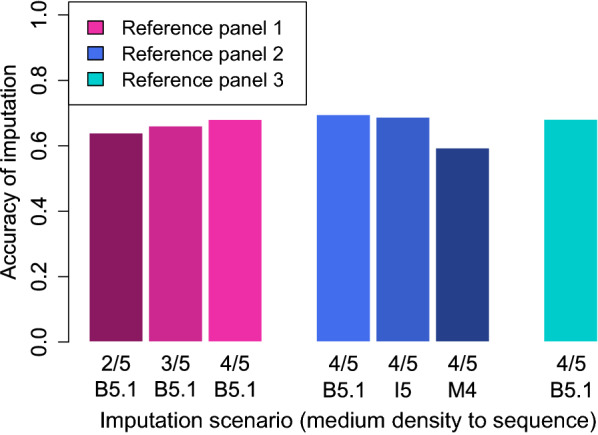
Accuracy of imputation from medium-density to sequence level for various imputation scenarios for the ECA3 chromosome. Imputation was performed for three reference panels (RP), including 65 (RP1), 175 (RP2) or 317 (RP3) horses. Imputation with RP1 was carried out for three panel sizes, including two (2/5), three (3/5) or four (4/5) fifths of the panel in the reference group. For imputation with RP2 and RP3, always four-fifth of the RP1 animals plus all additional animals of RP2 or RP3 were included. Imputation with RP2 was performed with three computer programs, Beagle 5.1 (B5.1), Impute 5 (I5) and Minimac 4 (M4). Otherwise, the software Beagle 5.1 was used for imputation, which in all cases was carried out for equine chromosome 3 (ECA3), as an example

The accuracy of imputation for ECA3 from medium marker density to sequence level increased from 0.64 to 0.68 stepwise as the size of RP1 increased from two- (26 animals) to four-fifths (52 animals) of the horses (Fig. 2). With respect to the reference group that was equal to four-fifths of RP1, addition of RP2 (162 animals) slightly improved imputation accuracy to 0.70, whereas the use of RP3 (304 animals) had almost no notable effect (Fig. 2). Using another software for imputation with RP2 did not improve the accuracy of imputation (Fig. 2), i.e., it was slightly reduced with Impute 5 (0.69), and dropped considerably with Minimac 4 (0.59). Imputation from high marker density to sequence level resulted in considerably higher values for imputation accuracy compared to imputation from medium marker density, with RP2 and RP3 (0.90) performing marginally better than RP1 (0.89). Stepwise imputation with RP1 did not improve the accuracy of imputation. The values for step one (0.61) and step two (0.59) were both lower than for direct imputation from medium density to sequence level using two-fifths of the animals of RP1 as the reference group (0.64). Excluding all markers with a DR2 value lower than 0.4 after the first step did not result in an improved accuracy for the second step, although the mean accuracy of the remaining markers after step one was 0.69. Likewise, imputation from medium to high marker density by using four-fifths of RP1 as the reference group resulted in lower values (0.66) than direct imputation to sequence level (0.68).

**Fig. 3:**
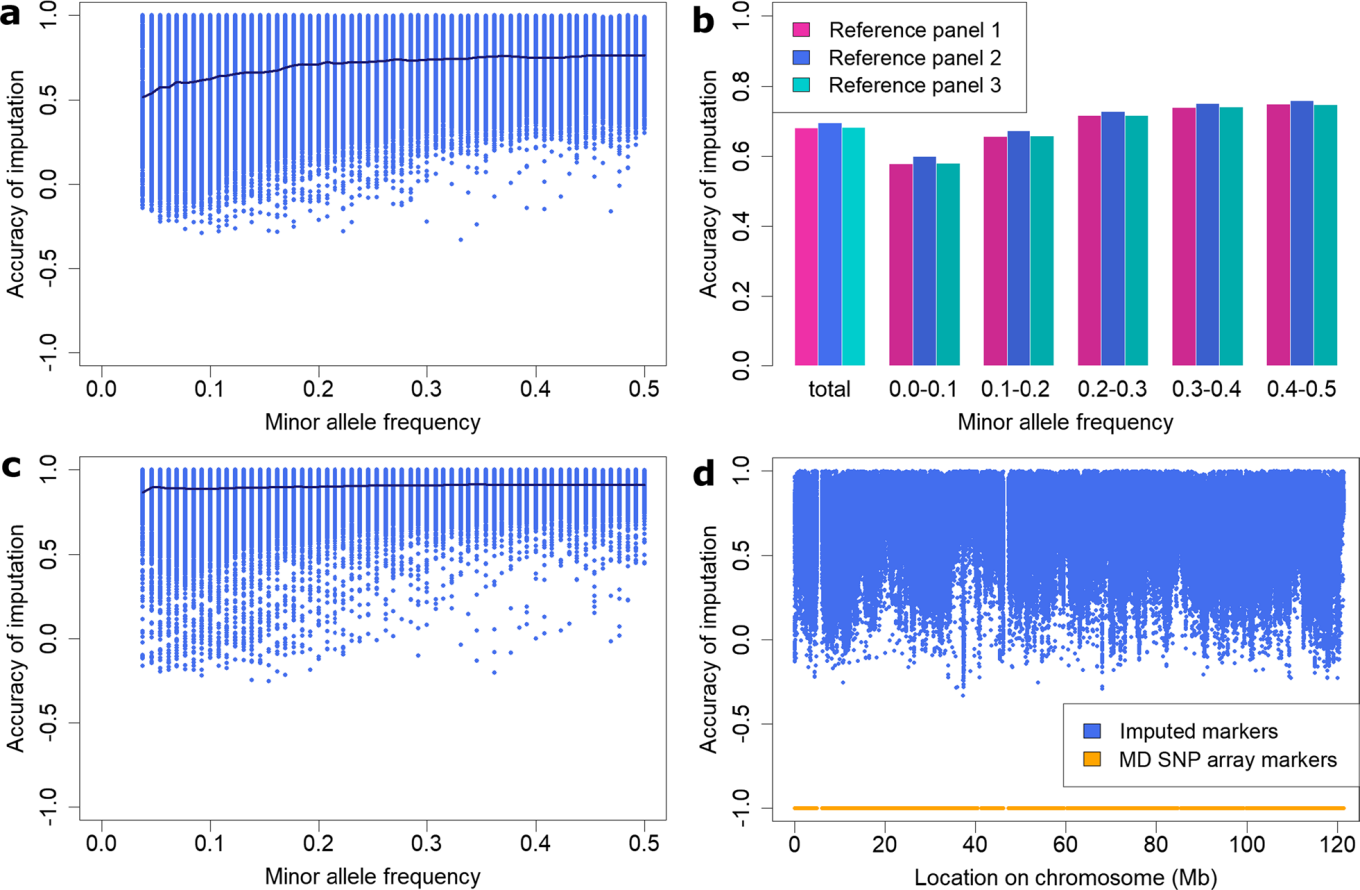
Accuracy of imputation to sequence level for the ECA3 chromosome. Imputation was performed for three reference panels (RP), including 52 (RP1), 162 (RP2) or 304 (RP3) horses, taking equine chromosome 3 (ECA3) as an example and using the software Beagle 5.1. **a** Accuracy of imputation plotted against minor allele frequency (MAF). Imputation from medium-density to sequence level applying RP2. The line in **a** and **c** is the average imputation accuracy per MAF. **b** Accuracy of imputation from medium-density versus MAF depending on the reference panel used for imputation. MAF categories ‘x–y’ stand for ‘x < MAF ≤ y’. **c** Accuracy of imputation from high-density to sequence level with RP2 plotted against MAF. **d** Accuracy of imputation from medium-density with RP2 plotted against the position on ECA3, with positions of the medium-density SNP array shown in orange

In general, the accuracy of imputation increased as the MAF of markers increased, especially for MAF lower than 0.2 (Fig. 3a). In comparison with RP1 and RP3, the use of RP2 improved the accuracy of imputation for markers with a low MAF (≤ 0.2) somewhat better than for markers with a higher MAF (Fig. 3b). For imputation from high marker density to sequence level, the dependency of the accuracy of imputation on MAF was less clear than for imputation from medium marker density to sequence level (Fig. 3c). Furthermore, the accuracy of imputation varied along ECA3, with reduced values in some regions and the most prominent reduction being between 37 and 38 Mb. In addition, two striking gaps were observed between 5 and 6 Mb and between 46 and 47 Mb, which corresponded to the absence of medium-density markers at those positions (Fig. 3d).

**Fig. 4:**
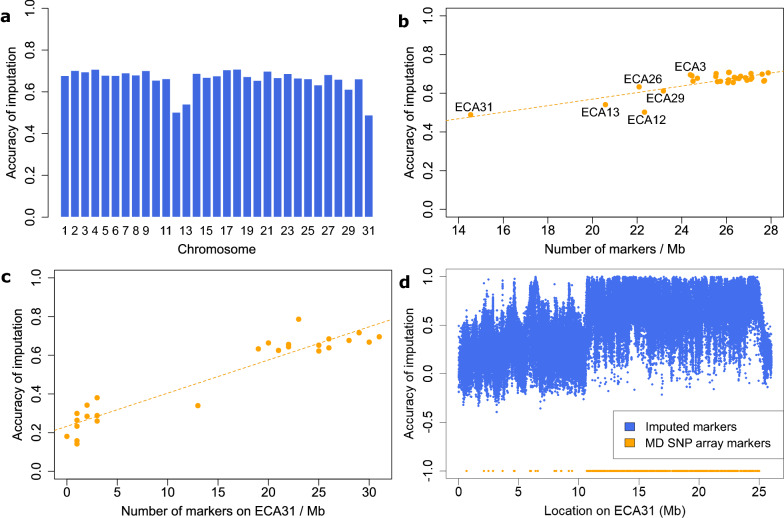
Accuracy of imputation from medium-density to sequence level. Imputation was performed using a reference panel of 162 horses (RP2) and the software Beagle 5.1. **a** Accuracy of imputation per chromosome. **b** Accuracy of imputation against marker density of the medium-density SNP array per chromosome. The dashed line indicates a linear regression model of y = 0.017x + 0.232 (adjusted R-squared: 0.669, p-value: 1.2 × 10^–8^). **c** Accuracy of imputation against marker density of the medium-density SNP array for the ECA31 chromosome. The dashed line indicates a linear regression model of y = 0.017  + 0.233 (adjusted R-squared: 0.896, p-value: 1.6 × 10^–13^). **d** Accuracy of imputation plotted against the position on ECA31, with positions of the medium-density (MD) SNP array shown in orange

Applying RP2 and Beagle 5.1, the mean accuracy of imputation from medium marker density to sequence level for the whole genome was 0.66 and ranged from 0.49 to 0.71 (Fig. 4a). Imputation accuracy was lower than 0.6 for the three ECA12, 13, and 31 chromosomes. For chromosomes with a higher marker density on the medium-density SNP array, imputation accuracy tended to be higher (Fig. 4b). Splitting ECA31 into 1-Mb segments and calculating the marker density of the medium-density SNP array and the accuracy of imputation for each segment separately showed an even more obvious correlation between marker density and imputation accuracy (Fig. 4c). Plotting the accuracy of imputation against the position on ECA31 showed that the accuracy of imputation was greatly reduced for regions with a low marker coverage, namely the first 11 and the last 1 Mb of this chromosome (Fig. 4d). Similar observations were made for other chromosomes (see Additional file 3: Figure S1). The correlation between MAF and imputation accuracy for the whole genome was very similar to the results for ECA3 as shown in Additional file 4: Figure S2.

### Analysis of information content

**Fig. 5:**
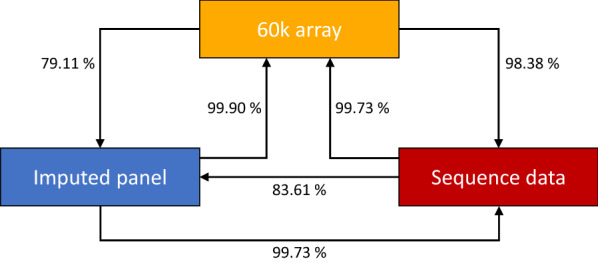
Monte Carlo analysis of variance for three interdependent datasets. Proportion of variation in the 60K data, the imputed panel and the sequence data that was explained by the variation in the two other respective datasets when using reference panel 2 and the Beagle 5.1 software

Since the results of the analysis of information content were very similar with both imputing methods, we present only the results obtained by combining the five separately imputed data panels into a joint set. Using RP2 with Beagle 5.1, the Monte Carlo analysis of variance indicated that the 60K data already explained 98.4% of the variation for the sequence panel (Fig. 5). This proportion was further increased by imputation, as the imputed panel included 99.7% of the variation of the sequence data. However, imputation did not only add information that was actually present in the real sequence data, since only 79.1% and 83.6% of the variation of the imputed panel were explained by the 60K and sequence data, respectively. As expected, both the imputed panel and the sequence data fully explained the 60K data, which indicates that no information was lost by imputation and thereby by increasing the marker density (Fig. 5).

When the size of the reference panel increased, the proportion of the variation of the imputed panel explained by the other panels decreased, which indicates that more noise was introduced to the model without any significant difference in the proportion of the variation of the sequence data. However, since this proportion reaches already 99.8%, this result should not be overinterpreted. The proportions of variation explained when using Impute 5 or Beagle 5.1 were very similar. However, when using Minimac 4, only 99.0% of the variation of the sequence data was explained by the imputed panel. A full list of the proportions of variation explained between the different panels is in Additional file 5: Table S2.

### Detection of putative lethal variants

Variant effect prediction of the biallelic sites with SnpEff annotated 53,656,734 effects on the variants, of which 59.7% were classified as intronic variants. In total, 23,148 variants were predicted to have a high impact on protein-coding sequences (see Additional file 6: Table S3) and 17,769 of those variants were not observed in the homozygous state. However, none of the variants showed a significant absence or reduction of homozygotes after correction for multiple testing.

## Discussion

### Accuracy of genotype imputation

To develop an optimal strategy for genotype imputation in German warmblood horses, we analysed the impact of several factors on the accuracy of imputation. Depending on the scenario, imputation accuracy for ECA3 ranged from 0.59 to 0.70 for the imputation from medium-density to sequence level. Considering the small number of available reference animals, especially of German warmblood horses, these results are comparable to those from studies on other livestock species that had similar preconditions, such as marker densities and the availability of reference animals [5, 11, 26, 52]. Only a few studies have evaluated the accuracy of imputation in horses and the reported estimates of imputation accuracy vary considerably depending on imputation strategies and preconditions [24–28].

### Size and composition of the reference panel

When the breed composition remained the same and only animals that are closely related to the study sample were included (imputation within RP1), increasing the size of the reference panel resulted in an improved accuracy of imputation. Similar observations have been made for the imputation in other livestock, such as cattle [5], sheep [21], chicken [52], and also horses [24]. To be precisely imputed, variants have to be present in the reference panel [48], and as the size of the reference panel increases, the number of haplotypes serving as a template for imputation increases [1].

It is less clear, whether more distantly-related animals, if available, should be added to the reference panel. We found that adding horses from breeds that were used as foundation stock for warmblood horses, such as Arabians [53] and Thoroughbreds [54], as well as breeds influenced by Thoroughbreds themselves, such as Standardbreds [54], improved the accuracy of imputation for ECA3 from 0.68 (RP1) to 0.70 (RP2). Conversely, including in RP3 all available horses, regardless of their genetic relationship to German warmblood horses, resulted in a drop in accuracy of imputation, which was similar to that obtained when using RP1, although the number of reference animals was almost six-fold greater than that of RP1.

Regarding the inclusion of multiple breeds in the reference panel, previous studies in various livestock populations have led to mixed conclusions. In several cases, using an extended multi-breed reference panel was advantageous compared to applying a single-breed panel in cattle [15, 17, 55] and sheep [2]. However, Hozé et al*.* [56] did not report any improvement of accuracy of imputation by adding individuals from other breeds to the reference panel. Likewise, contradictory observations have been made in horses. For three horse breeds, within-breed imputation turned out to be slightly more advantageous compared to imputation using a reference panel of various breeds, although the latter included considerably more animals than the reference panels consisting of only one breed [28]. Alternatively, for three other horse breeds, a large mixed-breed reference panel was shown to perform better than a small within-breed reference panel [24].

The question of how to select the animals to be included in the reference panel was recently addressed in detail for a panel of diverse chicken breeds [23]. The results demonstrated that error rates tended to decrease when subpopulations that showed shorter genetic distances to the study samples were included in the reference panel, while they increased when less related subpopulations were included. Hence, as stated by the aforementioned authors [23], optimisation of the accuracy of imputation with respect to reference panel composition is a trade-off between ensuring a sufficient large representation of genetic diversity of the animals and the introduction of unnecessary noise through the inclusion of genetically distant animals. An accurate determination of the genetic relationship between individuals is not easily obtained and the availability of WGS data for specific horse breeds is limited. Therefore, our findings are consistent with those of other studies and suggest that all the horses not only from the particular breed of interest but also from related breeds should be included in the reference panel for imputation but that the horses from breeds that are genetically distant to the study population should be omitted.

### Imputation software

The choice of the imputation software noticeably affected the accuracy of imputation, as already shown in other studies (e.g., [15, 26]). While Beagle 5.1 and Impute 5 performed similarly, using Minimac 4 resulted in a considerable drop in accuracy of imputation. All three programs are based on a Hidden Markov model [13, 48, 49] as first proposed for genotype imputation by Li and Stephens [57]. Since most available imputation tools were initially developed and optimised for outbred human populations [23], both Beagle 5.1 and Impute 5 parameter settings were adapted to improve their performance with livestock data. Both software tools allow to adapt the size of N_e_ while Minimac 4 does not and was therefore used without adapting this parameter. According to the results of a study in chicken and maize, adjustment of N_e_ size in the Beagle tool is of essential importance for accurate imputation in populations that have a low level of genetic diversity [23]. Hence, it cannot be excluded that the absence of an option to adapt N_e_ in Minimac 4 might be partly responsible for the markedly lower accuracy achieved with this tool.

### SNP array density

Imputation from high-density to sequence level was considerably more accurate than imputation from medium-density, as previously reported in cattle [5, 20] and chicken [52]. In contrast to imputation from medium-density, both RP2 and RP3 slightly outperformed RP1 in the case of imputation from high-density. The improved accuracy of imputation using a reference panel including all available horse breeds may be caused, to some extent, by the design of the different genotyping arrays. Contrary to previous medium-density arrays, the high-density Axiom Equine Genotyping Array (Axiom MNEc670; Applied Biosystems) was explicitly designed for accurate imputation to higher marker densities, especially the two million SNPs of the MNEc2M array, and to sequence level [58]. Furthermore, more data from other horse breeds were included in the development of the high-density than the medium-density arrays [27, 59].

### Two-step vs. one-step imputation

In contrast to studies in cattle [5, 19, 20], sheep [21], and chicken [52], stepwise imputation did not improve the accuracy of imputation but instead resulted in a drop of the accuracy of imputation. Increased error rates, which occurred in the first step of imputation from medium to high marker density, likely led to an overall reduction in accuracy through multiplication in the second step. Apparently, filtering out also poorly imputed markers prior to the second step of imputation could not improve the mean accuracy of the first step sufficiently to avoid this problem. This phenomenon was also described by Korkuć et al*.* [22] in cattle, when the reference panel for imputation to the intermediate level was small. These authors showed that only when large numbers of reference animals (i.e., 2145) were available for the first step of imputation (50K to 700K) and a few reference animals (30) were used for the second step (700K to sequence level), did the two-step imputation outperform the one-step approach.

In sheep, two-step imputation was performed with a very large number of reference animals (17,000) for the first step of imputation from 5K to 50K and a smaller reference panel (500) for the second step from 50K to 600K [21]. In this case, stepwise imputation outperformed direct imputation from 5K to 600K using a reference panel of 500 animals. Nevertheless, imputation from 5K to 50K was less accurate than from 5K to 600K when including the same number of animals in the reference panel. The same phenomenon was observed in this study when comparing imputation from 60K to 670K with imputation from 60K to sequence level, both when using two-fifths or four-fifths of RP1 as the reference group.

Accordingly, two-step imputation has proven to be particularly beneficial when a large reference panel is available for the first step of the procedure [19, 21, 22]. Therefore, the preconditions for two-step imputation were not optimal in our study, since the sizes of the reference panels for both imputation steps were identical. Here, stepwise imputation appeared to multiply the number of errors from step to step instead of being advantageous. However, in cattle, a similar approach with equally-sized reference panels resulted in improved accuracies for two-step imputation compared to direct imputation from 50K to sequence level [5].

### Chromosome position and minor allele frequency

The accuracy of imputation varied between chromosomes and chromosomal regions, with local drops and gaps that were possibly caused by genome assembly errors. For instance, the absence of SNP array markers and imputed variants in two regions of ECA3 are possibly due to assembly errors or structural variants that result in poor resolution of these regions. We observed reduced sequencing coverage for these two regions compared to that of the whole chromosome. A reduced accuracy of imputation for certain chromosomal regions or for whole chromosomes is usually associated with a reduced marker density of the medium-density SNP array in the respective regions. The three chromosomes with an accuracy of imputation lower than 0.6, i.e., ECA12, 13, and 31, displayed large regions with a greatly reduced marker coverage, the most prominent example being on ECA31. A notably reduced accuracy of imputation from the medium-density arrays was previously reported for ECA12 [26, 28, 60] and ECA13 [26, 60] and a slightly reduced accuracy for ECA31 was also found for Arabian horses [28]. Another reason for a low imputation accuracy in specific regions of the genome could be the presence of loci with a high level of sequence similarity and/or of highly polymorphic loci, such as in the major histocompatibility complex (MHC) region [61]. Spanning ~ 4 Mb on ECA20 [62], the MHC region was found to have a reduced accuracy of imputation (0.58) compared to the whole chromosome (0.65) or the whole genome (0.66).

Imputation accuracy improved as the MAF of markers increased, as previously reported [18, 52]. Compared to RP1 or RP3, the use of RP2 tended to be particularly advantageous for the imputation of markers with a MAF lower than 0.2. The observation that a multi-breed reference panel was especially beneficial for the imputation of markers with a low MAF was also made in cattle [17, 63], because alleles that are rare in one population may have higher frequencies in other populations [5]. In our cross-validation procedure, variants that were monomorphic in at least one of the validation groups could not be considered. Therefore, a number of rare SNPs were excluded from the analysis. As markers with a low MAF tend to be imputed less precisely, the overall imputation accuracy might be overestimated.

### Analysis of information content

Our analysis on information content indicates that any results obtained for specific imputed markers should be taken with extreme caution. At least for our data panel that covers a diverse horse population, imputation added noise rather than actual information that was not already present in the 60K data. Thus, target loci or regions should be subsequently validated by controlling the imputation accuracy, e.g., if provided by the imputation software, by controlling the DR2 values for the target markers [64] or by subsequent sequencing of the target area. Note that our analysis of the information content does not take into account that imputation can facilitate the detection of genomic regions that are associated with certain traits of interest because it increases the marker resolution even if no additional variation is added to the dataset. These observations are also in line with previous analyses from other studies, as only minor gains are obtained when increasing the marker density in genomic prediction [65, 66], while the resolution of GWAS tends to improve and more hits are obtained when using imputation [12, 67].

### Identification of putative lethal variants

By analysing WGS data on 317 horses from various breeds, we were unable to detect any high-impact variants that indicated a significant absence or reduction of homozygotes. In cattle, several potentially deleterious haplotypes have been discovered based on the principle of missing homozygosity [31–34]. On the contrary, in horses, this approach to detect lethal variants has only been used to a limited extent [37–39]. A major shortcoming of such analyses is that, especially for variants with a low frequency, a large sample size is necessary to statistically prove missing homozygosity. In our study, the sample size (317 horses) was rather small and included multiple breeds. Given the sample size and the number of identified high-impact variants, in our scenario, a MAF higher than 0.2 would be necessary to statistically prove missing homozygosity using the rules of Hardy-Weinberg equilibrium. However, the highest allele frequency of a high-impact variant for which no homozygotes were observed in our dataset was 0.168. Conversely, a sample size of 1299 animals would be necessary to statistically prove missing homozygosity for a variant with a MAF of 0.1. Hence, in our study, the preconditions to detect potentially lethal variants based on missing homozygosity were not optimal.

Nevertheless, we were able to validate the occurrence of variants in our dataset that had previously been reported to be deleterious. For example, a 5-bp deletion in the *PRKDC* (*protein kinase, DNA-activated, catalytic subunit*) gene was present among our high-impact variants in the heterozygous state in three Arabians and one German riding pony, but did not occur in the homozygous state (NC_009152.3:g.36395752_36395756del). This frameshift mutation results in the truncation of the protein and causes the severe combined immunodeficiency (SCID) disease in horses [68]. SCID is an autosomal recessive disorder in Arabians [69, 70], which is usually lethal in the first 5 months of life due to affected foals being unable to induce an adequate antigen-specific immune response [71]. Furthermore, our dataset contained a variant that had previously been suspected to be recessive lethal – a nonsense mutation (rs395871388) in the *PALB2* (*partner and localizer of BRCA2*) gene. This variant had already been reported by Jagannathan et al*.* [39] in two horses and was present in our variant call set in seven horses in the heterozygous state but did not occur in the homozygous state. Two of the horses were also included in the aforementioned study [39]. *PALB2* has been shown to be essential for early embryogenesis in mice [72].

To be able to statistically prove missing homozygosity for the identified high-impact variants, our results should be validated and further investigated in a larger study cohort. However, the availability of WGS data from horses is currently limited. An expansion of the sample size for German warmblood horses is expected soon due to the establishment of a large reference population of 5000 animals with genome-wide SNP genotypes and phenotypes to implement genomic selection [29]. Although marker data can be used to map causal variants to genomic regions, to identify the causative mutations, sequence data are required. In contrast to SNP arrays, sequence data contain the causal variants [6]. Using imputed sequence-level data for the discovery of putative lethal mutations has its limitations compared to the use of WGS data. Lethal variants have to be present in the reference panel in order to be correctly imputed and subsequently identified. This is of highest importance for recently emerged variants, which may not exist in the reference population at all, or for which the haplotype carrying the mutation might be present in two forms, with and without the new mutation. This can lead to incorrect imputation results and therefore result in missing lethal variants. Nevertheless, genotype imputation is a cost-effective method to obtain sequence-level data for large cohorts of animals, such as the aforementioned reference population of warmblood horses. When the availability of WGS data for a species is limited, the restricted sample size may not allow for the statistical verification of missing homozygosity. In this case, genotype imputation can improve the detection of lethal variants based on missing homozygosity by increasing the number of markers compared to the use of SNP array data.

## Conclusions

The accuracy of imputation from SNP array data to sequence level in German warmblood horses was influenced by several factors: size and composition of the reference panel, marker density of the genotyping array, imputation software and MAF of the imputed markers. Knowing these effects allows adjusting the strategy of imputation in order to achieve a maximum accuracy of imputation even in datasets where true genotypes are not known. Our reference panel is a valuable resource for the application of genotype imputation in horses. By genotype imputation, the limited amount of currently available WGS data from horses can be supplemented to boost the power of downstream analyses. We have also shown that imputation does not only add real variation to the dataset but also noise. Thus, imputed genotypes need to be approached with caution and require additional validation. In this study, no high-impact variants showing a significant absence or reduction of homozygotes could be detected. However, the sample was diverse and of limited size and imputed sequence-level genotypes could be used to improve the identification of embryonic lethal variants from genomic data.

## Supplementary Information

**Additional file 1: Table S1.** Horse genomes included in the study. Accession and description of whole-genome sequence data from all horses included in the study. Information was obtained from the NCBI Sequence Read Archive (SRA). The classification of the horses into three reference panel groups based on multidimensional scaling or the reason for excluding the respective genomes from the study, if applicable (excluded genomes are highlighted in grey), are also indicated.

**Additional file 2.** R script to calculate the accuracy of imputation. This R script was used to calculate the accuracy of imputation as the correlation between observed genotypes and estimated alternate allele dosages across all samples.

**Additional file 3: Figure S1.** Accuracy of imputation from medium-density to sequence level per chromosome. Accuracy of imputation plotted against the position on the equine chromosomes, with positions of the medium-density SNP array shown in orange. Imputation was performed using a reference panel of 162 horses (RP2) and the software Beagle 5.1.

**Additional file 4: Figure S2.** Accuracy of genome-wide imputation from medium-density to sequence level against minor allele frequency. Imputation was performed using a reference panel of 162 horses (RP2) and the software Beagle 5.1. The line is the average imputation accuracy per minor allele frequency.

**Additional file 5: Table S2.** Monte Carlo analysis of variance for three interdependent datasets. Proportion of the variation in the 60K data, the imputed panel and the sequence data that was explained by the variation in the two other respective datasets, depending on the reference panel (RP) and imputation software used.

**Additional file 6: Table S3.** High-impact variants in the variants call set of 317 horses. High-impact variants identified by variant effect prediction were tested for a significant absence or reduction of homozygotes using a binomial test under the assumptions of Hardy-Weinberg equilibrium. The obtained p-values were adjusted for multiple testing using Bonferroni correction. Variants that affect more than one gene or have different effects on different isoforms of a protein are listed multiple times, once for each gene/isoform. However, for significance testing, each variant was only considered once.

## Data Availability

The WGS data of all horses included in the study were acquired from the European Nucleotide Archive (ENA) at EMBL-EBI (https://www.ebi.ac.uk/ena/browser/view) under the accession numbers stated in Additional file 1: Table S1. The horse genome assembly used throughout the study was EquCab3.0 (GCA_002863925.1) obtained from Ensembl (ftp://ftp.ensembl.org/pub/release-100/fasta/equus_caballus/dna/Equus_caballus.EquCab3.0.dna.toplevel.fa.gz). Likewise, the known sites’ database dbSNP build 151 was acquired from Ensembl (ftp://ftp.ensembl.org/pub/release-101/variation/vcf/equus_caballus/equus_caballus.vcf.gz).

## References

[1] Das S, Abecasis GR, Browning BL (2018). Genotype imputation from large reference panels. Annu Rev Genomics Hum Genet.

[2] Bolormaa S, Chamberlain AJ, Khansefid M, Stothard P, Swan AA, Mason B (2019). Accuracy of imputation to whole-genome sequence in sheep. Genet Sel Evol.

[3] Meuwissen T, Goddard M (2010). Accurate prediction of genetic values for complex traits by whole-genome resequencing. Genetics.

[4] van Binsbergen R, Calus MPL, Bink MCAM, van Eeuwijk FA, Schrooten C, Veerkamp RF (2015). Genomic prediction using imputed whole-genome sequence data in Holstein Friesian cattle. Genet Sel Evol.

[5] van Binsbergen R, Bink MC, Calus MP, van Eeuwijk FA, Hayes BJ, Hulsegge I (2014). Accuracy of imputation to whole-genome sequence data in Holstein Friesian cattle. Genet Sel Evol.

[6] Xiang R, MacLeod IM, Daetwyler HD, de Jong G, O'Connor E, Schrooten C (2021). Genome-wide fine-mapping identifies pleiotropic and functional variants that predict many traits across global cattle populations. Nat Commun.

[7] Ni G, Cavero D, Fangmann A, Erbe M, Simianer H (2017). Whole-genome sequence-based genomic prediction in laying chickens with different genomic relationship matrices to account for genetic architecture. Genet Sel Evol.

[8] Frischknecht M, Meuwissen THE, Bapst B, Seefried FR, Flury C, Garrick D (2018). Short communication: genomic prediction using imputed whole-genome sequence variants in Brown Swiss Cattle. J Dairy Sci.

[9] Brøndum RF, Su G, Janss L, Sahana G, Guldbrandtsen B, Boichard D (2015). Quantitative trait loci markers derived from whole genome sequence data increases the reliability of genomic prediction. J Dairy Sci.

[10] Moghaddar N, Khansefid M, van der Werf JHJ, Bolormaa S, Duijvesteijn N, Clark SA (2019). Genomic prediction based on selected variants from imputed whole-genome sequence data in Australian sheep populations. Genet Sel Evol.

[11] van den Berg S, Vandenplas J, van Eeuwijk FA, Bouwman AC, Lopes MS, Veerkamp RF (2019). Imputation to whole-genome sequence using multiple pig populations and its use in genome-wide association studies. Genet Sel Evol.

[12] Marchini J, Howie B (2010). Genotype imputation for genome-wide association studies. Nat Rev Genet.

[13] Rubinacci S, Delaneau O, Marchini J (2020). Genotype imputation using the Positional Burrows Wheeler Transform. PLoS Genet.

[14] de los Campos G, Pook T, Gonzalez-Reymundez A, Simianer H, Mias G, Vazquez AI (2020). ANOVA-HD: analysis of variance when both input and output layers are high-dimensional. PLoS ONE.

[15] Daetwyler HD, Capitan A, Pausch H, Stothard P, van Binsbergen R, Brøndum RF (2014). Whole-genome sequencing of 234 bulls facilitates mapping of monogenic and complex traits in cattle. Nat Genet.

[16] Wang Y, Lin G, Li C, Stothard P (2016). Genotype imputation methods and their effects on genomic predictions in cattle. Springer Sci Rev.

[17] Bouwman AC, Veerkamp RF (2014). Consequences of splitting whole-genome sequencing effort over multiple breeds on imputation accuracy. BMC Genet.

[18] Butty AM, Sargolzaei M, Miglior F, Stothard P, Schenkel FS, Gredler-Grandl B (2019). Optimizing selection of the reference population for genotype imputation from array to sequence variants. Front Genet.

[19] Khatkar MS, Moser G, Hayes BJ, Raadsma HW (2012). Strategies and utility of imputed SNP genotypes for genomic analysis in dairy cattle. BMC Genomics.

[20] VanRaden PM, Null DJ, Sargolzaei M, Wiggans GR, Tooker ME, Cole JB (2013). Genomic imputation and evaluation using high-density Holstein genotypes. J Dairy Sci.

[21] Ventura RV, Miller SP, Dodds KG, Auvray B, Lee M, Bixley M (2016). Assessing accuracy of imputation using different SNP panel densities in a multi-breed sheep population. Genet Sel Evol.

[22] Korkuć P, Arends D, Brockmann GA (2019). Finding the optimal imputation strategy for small cattle populations. Front Genet.

[23] Pook T, Mayer M, Geibel J, Weigend S, Cavero D, Schoen CC (2020). Improving imputation quality in BEAGLE for crop and livestock data. G3 (Bethesda)..

[24] McCoy AM, McCue ME (2014). Validation of imputation between equine genotyping arrays. Anim Genet.

[25] Corbin LJ, Kranis A, Blott SC, Swinburne JE, Vaudin M, Bishop SC (2014). The utility of low-density genotyping for imputation in the Thoroughbred horse. Genet Sel Evol.

[26] Frischknecht M, Neuditschko M, Jagannathan V, Drögemüller C, Tetens J, Thaller G (2014). Imputation of sequence level genotypes in the Franches-Montagnes horse breed. Genet Sel Evol.

[27] Schaefer RJ, Schubert M, Bailey E, Bannasch DL, Barrey E, Bar-Gal GK (2017). Developing a 670k genotyping array to tag ~2M SNPs across 24 horse breeds. BMC Genomics.

[28] Chassier M, Barrey E, Robert C, Duluard A, Danvy S, Ricard A (2018). Genotype imputation accuracy in multiple equine breeds from medium- to high-density genotypes. J Anim Breed Genet.

[29] Vosgerau S, Krattenmacher N, Falker-Gieske C, Blaj I, Seidel A, Wobbe M, et al. Towards genomic selection in German warmblood horses. In: Proceedings of the 71st Annual Meeting of the European Federation of Animal Science: 1–4 December 2020; Wageningen. Virtual; 2020.

[30] VanRaden PM, Olson KM, Null DJ, Hutchison JL (2011). Harmful recessive effects on fertility detected by absence of homozygous haplotypes. J Dairy Sci.

[31] Fritz S, Capitan A, Djari A, Rodriguez SC, Barbat A, Baur A (2013). Detection of haplotypes associated with prenatal death in dairy cattle and identification of deleterious mutations in *GART*, *SHBG* and *SLC37A2*. PLoS ONE.

[32] Pausch H, Schwarzenbacher H, Burgstaller J, Flisikowski K, Wurmser C, Jansen S (2015). Homozygous haplotype deficiency reveals deleterious mutations compromising reproductive and rearing success in cattle. BMC Genomics.

[33] Hoff JL, Decker JE, Schnabel RD, Taylor JF (2017). Candidate lethal haplotypes and causal mutations in Angus cattle. BMC Genomics.

[34] Wu X, Mesbah-Uddin M, Guldbrandtsen B, Lund MS, Sahana G (2020). Novel haplotypes responsible for prenatal death in Nordic Red and Danish Jersey cattle. J Dairy Sci.

[35] Derks MFL, Megens H-J, Bosse M, Lopes MS, Harlizius B, Groenen MAM (2017). A systematic survey to identify lethal recessive variation in highly managed pig populations. BMC Genomics.

[36] Derks MFL, Gjuvsland AB, Bosse M, Lopes MS, van Son M, Harlizius B (2019). Loss of function mutations in essential genes cause embryonic lethality in pigs. PLoS Genet.

[37] Schrimpf R, Gottschalk M, Metzger J, Martinsson G, Sieme H, Distl O (2016). Screening of whole genome sequences identified high-impact variants for stallion fertility. BMC Genomics.

[38] Todd ET, Thomson PC, Hamilton NA, Ang RA, Lindgren G, Viklund Å (2020). A genome-wide scan for candidate lethal variants in Thoroughbred horses. Sci Rep.

[39] Jagannathan V, Gerber V, Rieder S, Tetens J, Thaller G, Drögemüller C (2019). Comprehensive characterization of horse genome variation by whole-genome sequencing of 88 horses. Anim Genet.

[40] McKenna A, Hanna M, Banks E, Sivachenko A, Cibulskis K, Kernytsky A (2010). The Genome Analysis Toolkit: a MapReduce framework for analyzing next-generation DNA sequencing data. Genome Res.

[41] van der Auwera GA, O'Connor BD (2020). Genomics in the cloud: using Docker, GATK, and WDL in Terra.

[42] Li H, Durbin R (2009). Fast and accurate short read alignment with Burrows-Wheeler transform. Bioinformatics.

[43] Nicolazzi EL, Caprera A, Nazzicari N, Cozzi P, Strozzi F, Lawley C (2015). SNPchiMp vol 3: Integrating and standardizing single nucleotide polymorphism data for livestock species. BMC Genomics.

[44] Danecek P, Bonfield JK, Liddle J, Marshall J, Ohan V, Pollard MO (2021). Twelve years of SAMtools and BCFtools. Gigascience.

[45] Chang CC, Chow CC, Tellier LC, Vattikuti S, Purcell SM, Lee JJ (2015). Second-generation PLINK: rising to the challenge of larger and richer datasets. Gigascience.

[46] R Core Team (2021). R: a language and environment for statistical computing.

[47] Browning SR, Browning BL (2007). Rapid and accurate haplotype phasing and missing-data inference for whole-genome association studies by use of localized haplotype clustering. Am J Hum Genet.

[48] Browning BL, Zhou Y, Browning SR (2018). A one-penny imputed genome from next-generation reference panels. Am J Hum Genet.

[49] Das S, Forer L, Schönherr S, Sidore C, Locke AE, Kwong A (2016). Next-generation genotype imputation service and methods. Nat Genet.

[50] Cingolani P, Platts A, Le Wang L, Coon M, Nguyen T, Wang L (2012). A program for annotating and predicting the effects of single nucleotide polymorphisms, SnpEff: SNPs in the genome of Drosophila melanogaster strain w1118; iso-2; iso-3. Fly (Austin).

[51] Cingolani P, Patel VM, Coon M, Nguyen T, Land SJ, Ruden DM, Lu X (2012). Using Drosophila melanogaster as a model for genotoxic chemical mutational studies with a new program. SnpSift Front Genet.

[52] Ye S, Yuan X, Lin X, Gao N, Luo Y, Chen Z (2018). Imputation from SNP chip to sequence: a case study in a Chinese indigenous chicken population. J Anim Sci Biotechnol.

[53] Wallner B, Vogl C, Shukla P, Burgstaller JP, Druml T, Brem G (2013). Identification of genetic variation on the horse y chromosome and the tracing of male founder lineages in modern breeds. PLoS ONE.

[54] Petersen JL, Mickelson JR, Cothran EG, Andersson LS, Axelsson J, Bailey E (2013). Genetic diversity in the modern horse illustrated from genome-wide SNP data. PLoS ONE.

[55] Rowan TN, Hoff JL, Crum TE, Taylor JF, Schnabel RD, Decker JE (2019). A multi-breed reference panel and additional rare variants maximize imputation accuracy in cattle. Genet Sel Evol.

[56] Hozé C, Fouilloux MN, Venot E, Guillaume F, Dassonneville R, Fritz S (2013). High-density marker imputation accuracy in sixteen French cattle breeds. Genet Sel Evol.

[57] Li N, Stephens M (2003). Modeling linkage disequilibrium and identifying recombination hotspots using single-nucleotide polymorphism data. Genetics.

[58] Schaefer RJ, McCue ME (2020). Equine genotyping arrays. Vet Clin North Am Equine Pract.

[59] McCue ME, Bannasch DL, Petersen JL, Gurr J, Bailey E, Binns MM (2012). A high density SNP array for the domestic horse and extant Perissodactyla: utility for association mapping, genetic diversity, and phylogeny studies. PLoS Genet.

[60] Pereira GL, Chud TC, Bernardes PA, Venturini GC, Chardulo LA, Curi RA (2017). Genotype imputation and accuracy evaluation in racing quarter horses genotyped using different commercial SNP panels. J Equine Vet Sci.

[61] Viļuma A, Mikko S, Hahn D, Skow L, Andersson G, Bergström TF (2017). Genomic structure of the horse major histocompatibility complex class II region resolved using PacBio long-read sequencing technology. Sci Rep.

[62] Sadeghi R, Moradi-Shahrbabak M, Miraei Ashtiani SR, Miller DC, Antczak DF (2018). MHC haplotype diversity in Persian Arabian horses determined using polymorphic microsatellites. Immunogenetics.

[63] Pausch H, MacLeod IM, Fries R, Emmerling R, Bowman PJ, Daetwyler HD (2017). Evaluation of the accuracy of imputed sequence variant genotypes and their utility for causal variant detection in cattle. Genet Sel Evol.

[64] Browning BL, Browning SR (2009). A unified approach to genotype imputation and haplotype-phase inference for large data sets of trios and unrelated individuals. Am J Hum Genet.

[65] Erbe M, Hayes BJ, Matukumalli LK, Goswami S, Bowman PJ, Reich CM (2012). Improving accuracy of genomic predictions within and between dairy cattle breeds with imputed high-density single nucleotide polymorphism panels. J Dairy Sci.

[66] Ober U, Ayroles JF, Stone EA, Richards S, Zhu D, Gibbs RA (2012). Using whole-genome sequence data to predict quantitative trait phenotypes in *Drosophila melanogaster*. PLoS Genet.

[67] Yan G, Qiao R, Zhang F, Xin W, Xiao S, Huang T (2017). Imputation-based whole-genome sequence association study rediscovered the missing QTL for lumbar number in Sutai pigs. Sci Rep.

[68] Shin EK, Perryman LE, Meek K (1997). A kinase-negative mutation of DNA-PK(CS) in equine SCID results in defective coding and signal joint formation. J Immunol.

[69] McGuire TC, Poppie MJ (1973). Hypogammaglobulinemia and thymic hypoplasia in horses: a primary combined immunodeficiency disorder. Infect Immun.

[70] Perryman LE, Torbeck RL (1980). Combined immunodeficiency of Arabian horses: confirmation of autosomal recessive mode of inheritance. J Am Vet Med Assoc.

[71] Perryman LE (2004). Molecular pathology of severe combined immunodeficiency in mice, horses, and dogs. Vet Pathol.

[72] Rantakari P, Nikkilä J, Jokela H, Ola R, Pylkäs K, Lagerbohm H (2010). Inactivation of Palb2 gene leads to mesoderm differentiation defect and early embryonic lethality in mice. Hum Mol Genet.

[73] Reich P, Falker-Gieske C, Tetens J. Identification of putative lethal variants using whole-genome sequence data from various horse breeds. In: Proceedings of the 38th International Society for Animal Genetics Virtual Conference: 26–30 July 2021; Virtual. https://www.isag.us/Docs/Proceedings/ISAG2021_Proceedings.pdf?v=20211015. Accessed 28 Oct 2021.

