# Supplementary material for: Development and validation of a horse reference panel for genotype imputation
Source: Genet Sel Evol. 2022 Jul 4;54:49. doi: 10.1186/s12711-022-00740-8 (PMC9252005; doi:10.1186/s12711-022-00740-8)
Supplement: Supplementary file 3 — Additional file 3: Figure S1. Accuracy of imputation from medium-density to sequence level per chromosome. Accuracy of imputation plotted against the position on the equine chromosomes, with positions of the medium-density SNP array shown in orange. Imputation was performed using a reference panel of 162 horses (RP2) and the software Beagle 5.1. [file 12711_2022_740_MOESM3_ESM.pdf]

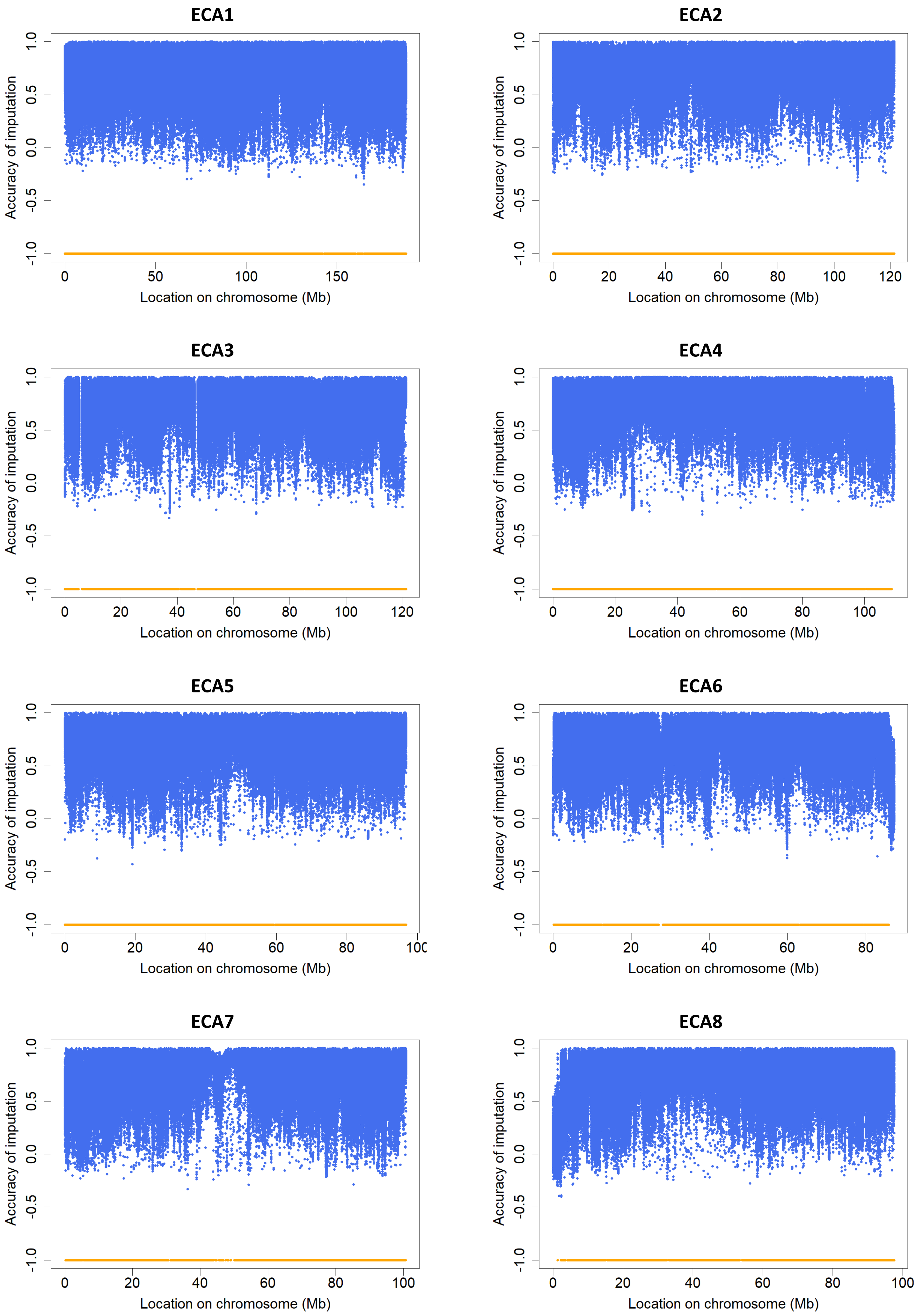

ECA1
Accuracy of imputation
1.0 0.5 0.0 -0.5 -1.0
0 50 100 150
Location on chromosome (Mb)
ECA2
Accuracy of imputation
1.0 0.5 0.0 -0.5 -1.0
0 20 40 60 80 100 120
Location on chromosome (Mb)
ECA3
Accuracy of imputation
1.0 0.5 0.0 -0.5 -1.0
0 20 40 60 80 100 120
Location on chromosome (Mb)
ECA4
Accuracy of imputation
1.0 0.5 0.0 -0.5 -1.0
0 20 40 60 80 100
Location on chromosome (Mb)
ECA5
Accuracy of imputation
1.0 0.5 0.0 -0.5 -1.0
0 20 40 60 80 100
Location on chromosome (Mb)
ECA6
Accuracy of imputation
1.0 0.5 0.0 -0.5 -1.0
0 20 40 60 80
Location on chromosome (Mb)
ECA7
Accuracy of imputation
1.0 0.5 0.0 -0.5 -1.0
0 20 40 60 80 100
Location on chromosome (Mb)
ECA8
Accuracy of imputation
1.0 0.5 0.0 -0.5 -1.0
0 20 40 60 80 100
Location on chromosome (Mb)

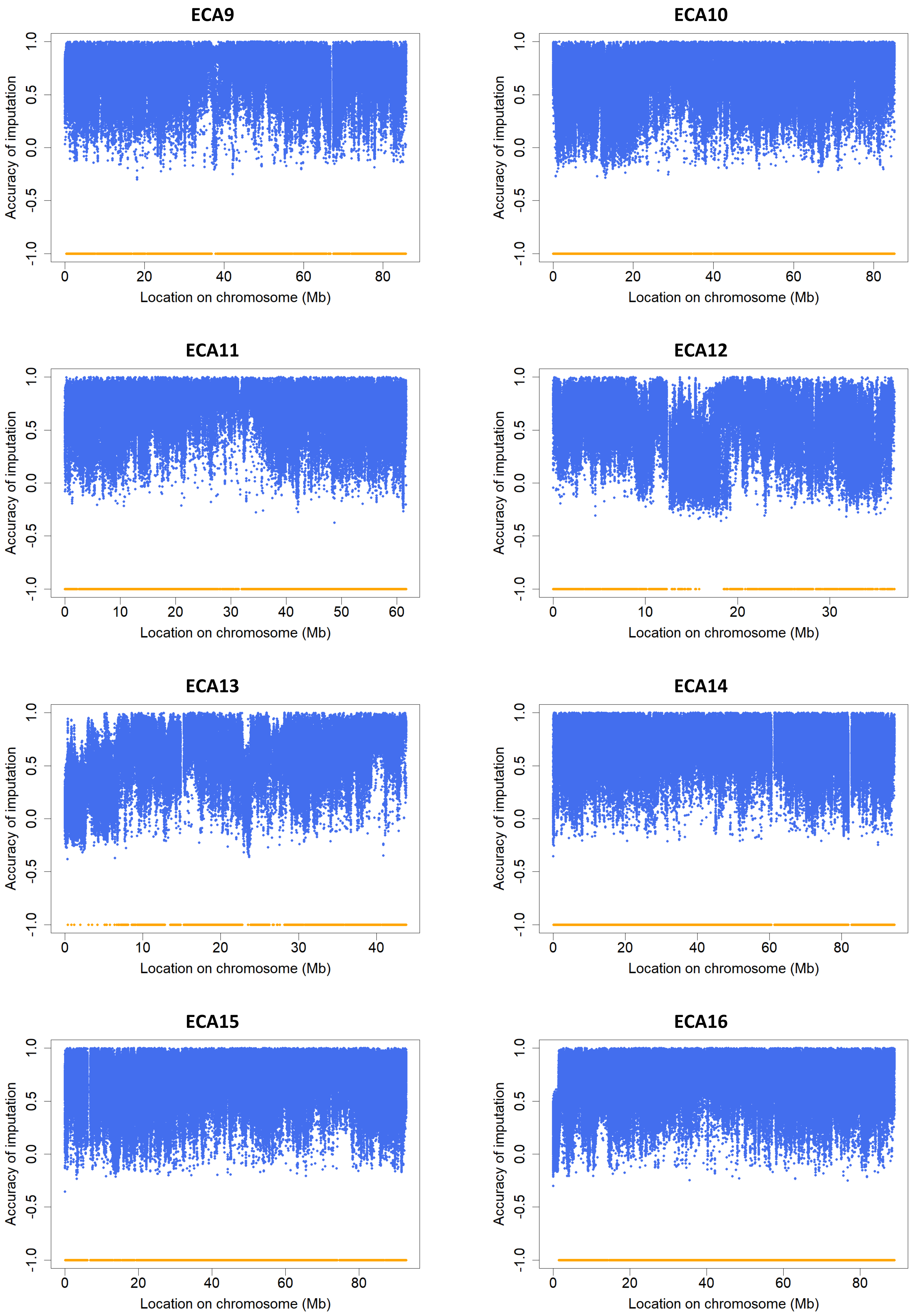
ECA9
ECA10
ECA11
ECA12
ECA13
ECA14
ECA15
ECA16
Accuracy of imputation
1.0
0.5
0.0
-0.5
-1.0
Location on chromosome (Mb)
0
10
20
30
40
50
60
80

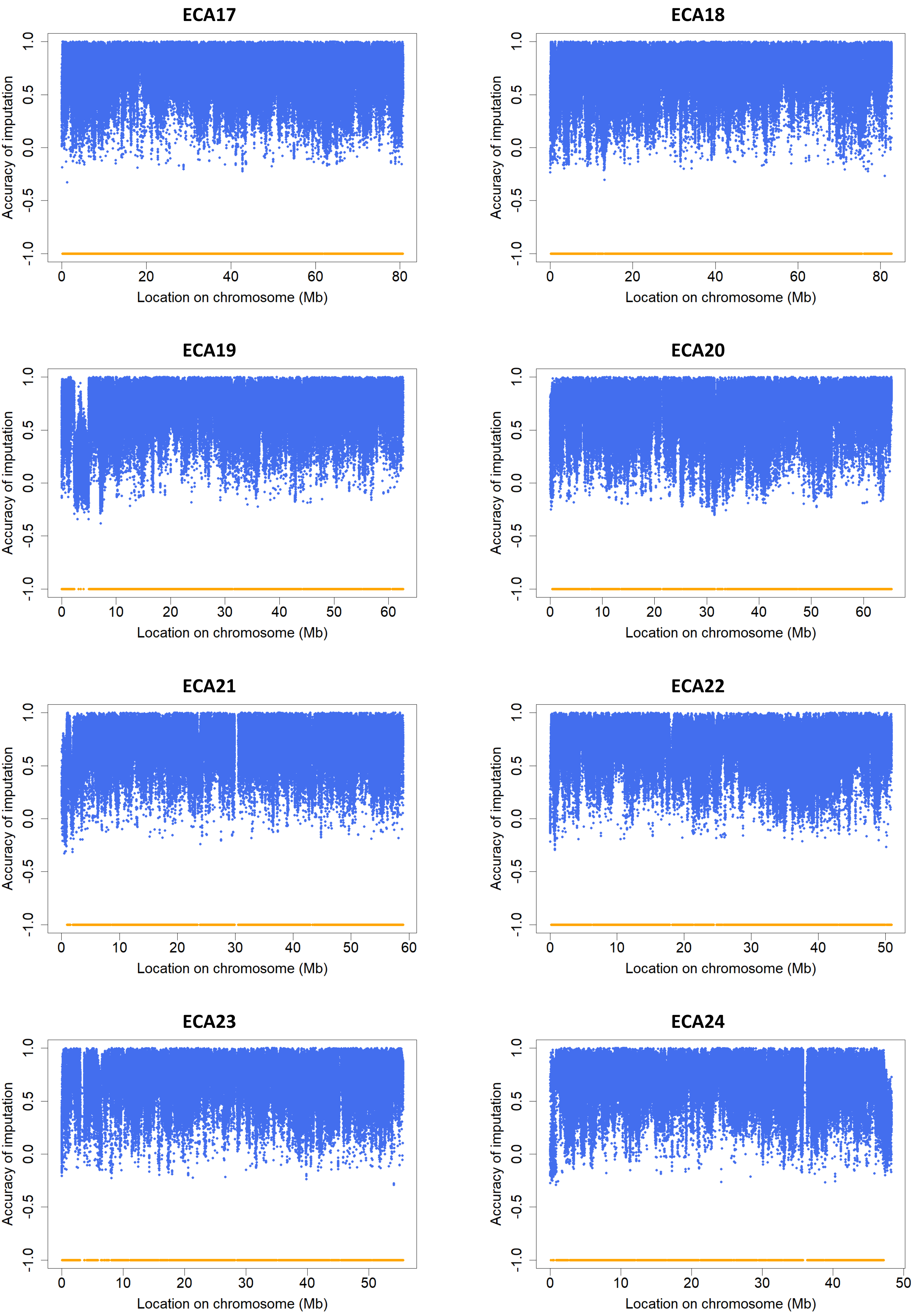
ECA17
Accuracy of imputation
1.0
0.5
0.0
-0.5
-1.0
0
20
40
60
80
Location on chromosome (Mb)
ECA18
Accuracy of imputation
1.0
0.5
0.0
-0.5
-1.0
0
20
40
60
80
Location on chromosome (Mb)
ECA19
Accuracy of imputation
1.0
0.5
0.0
-0.5
-1.0
0
10
20
30
40
50
60
Location on chromosome (Mb)
ECA20
Accuracy of imputation
1.0
0.5
0.0
-0.5
-1.0
0
10
20
30
40
50
60
Location on chromosome (Mb)
ECA21
Accuracy of imputation
1.0
0.5
0.0
-0.5
-1.0
0
10
20
30
40
50
60
Location on chromosome (Mb)
ECA22
Accuracy of imputation
1.0
0.5
0.0
-0.5
-1.0
0
10
20
30
40
50
Location on chromosome (Mb)
ECA23
Accuracy of imputation
1.0
0.5
0.0
-0.5
-1.0
0
10
20
30
40
50
Location on chromosome (Mb)
ECA24
Accuracy of imputation
1.0
0.5
0.0
-0.5
-1.0
0
10
20
30
40
50
Location on chromosome (Mb)

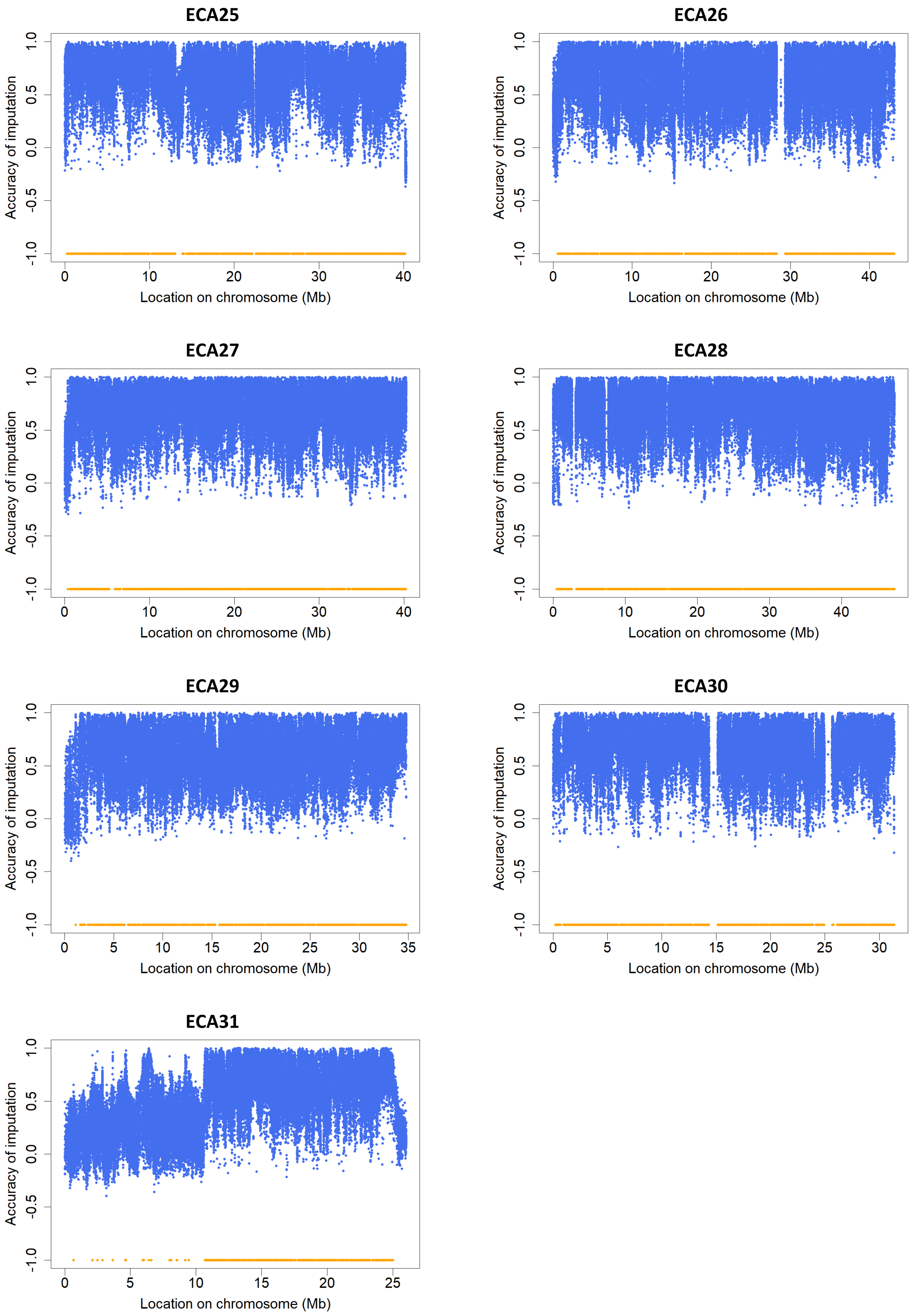
ECA25
ECA26
ECA27
ECA28
ECA29
ECA30
ECA31
Accuracy of imputation
1.0
0.5
0.0
-0.5
-1.0
Location on chromosome (Mb)
0
5
10
15
20
25
30
35
40
