# Supplementary figures and images for: Development and validation of a horse reference panel for genotype imputation
Source: Genet Sel Evol. 2022 Jul 4;54:49. doi: 10.1186/s12711-022-00740-8 (PMC9252005; doi:10.1186/s12711-022-00740-8)

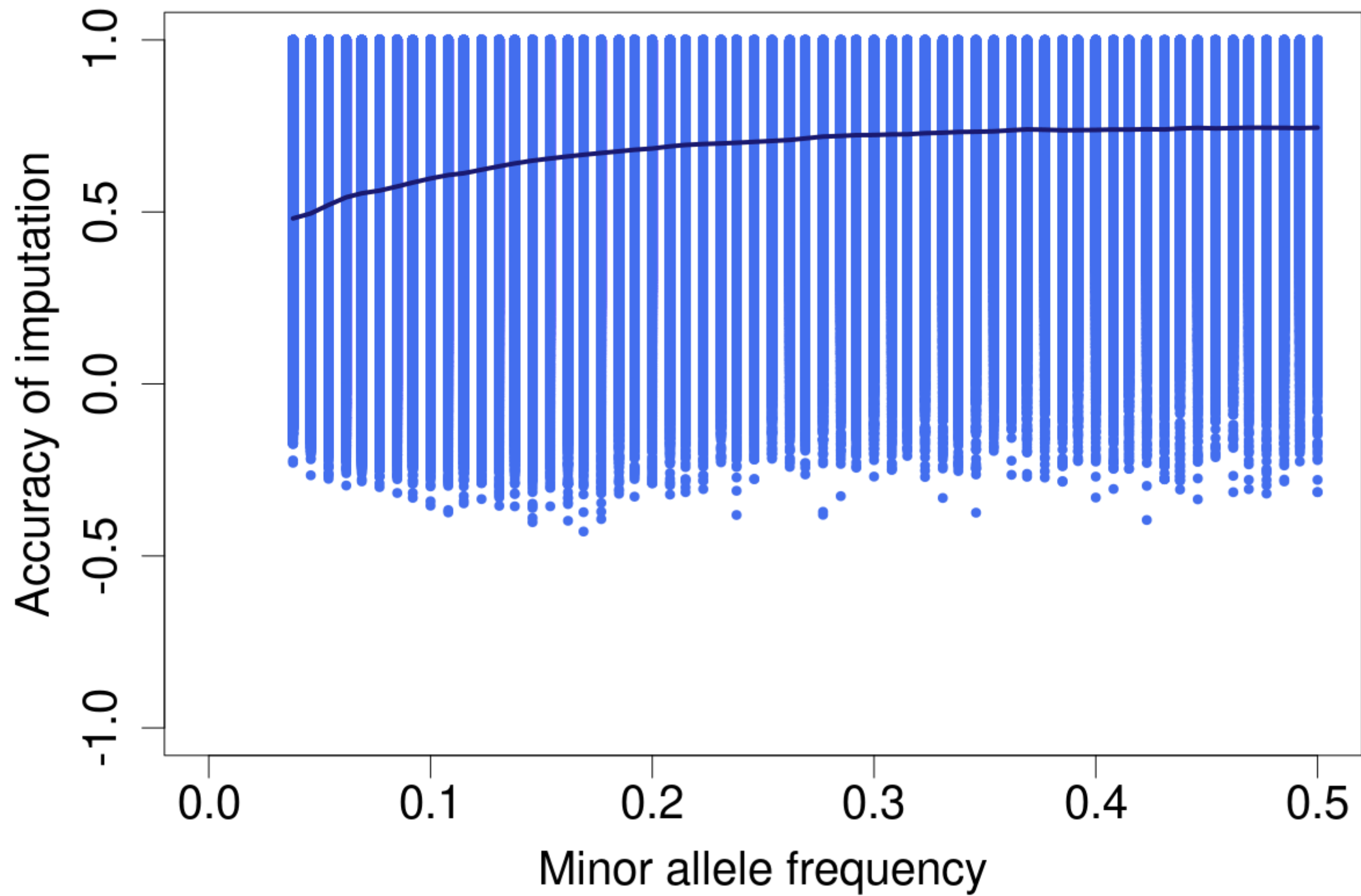

Supplement: Supplementary file 4 — Additional file 4: Figure S2. Accuracy of genome-wide imputation from medium-density to sequence level against minor allele frequency. Imputation was performed using a reference panel of 162 horses (RP2) and the software Beagle 5.1. The line is the average imputation accuracy per minor allele frequency. [file 12711_2022_740_MOESM4_ESM.pdf]
